# A Woman with Unilateral Rash and Fever: Cellulitis in the Setting of Lymphedema

**DOI:** 10.1155/2015/252495

**Published:** 2015-06-11

**Authors:** Melissa Joseph, Marissa Camilon, Tarina Kang

**Affiliations:** Department of Emergency Medicine, LAC+USC Medical Center, 1200 N. State Street, Los Angeles, CA 90033, USA

## Abstract

Cellulitis in the setting of lymphedema is an uncommon but clinically important presentation to the emergency department. Stagnant lymph is an ideal medium for bacterial growth and progression can be rapid due to decreased ability to fight infection in the affected area. Infections are commonly caused by gram-positive cocci, though blood cultures are often negative. Treatment should be aimed at rapid initiation of antibiotics targeting these species. There may be a role for antibiotic prophylaxis in recurrent cases.

## 1. Introduction

Cellulitis is a common presenting complaint to the emergency department (ED). Lymphedema cellulitis is a less common presentation of cellulitis, but it is important to recognize due to its aggressive onset, potential for recurrence, and increased risk of treatment failure [1, 2]. Patients with damaged or surgically removed lymphatic beds are at increased risk for cellulitis secondary to impairment of both lymphatic flow and elimination of phagocytosed bacteria [1, 3]. We present a case of cellulitis in the setting of previous axillary lymph node dissection and lymphedema.

## 2. Case

**Figure 1:**
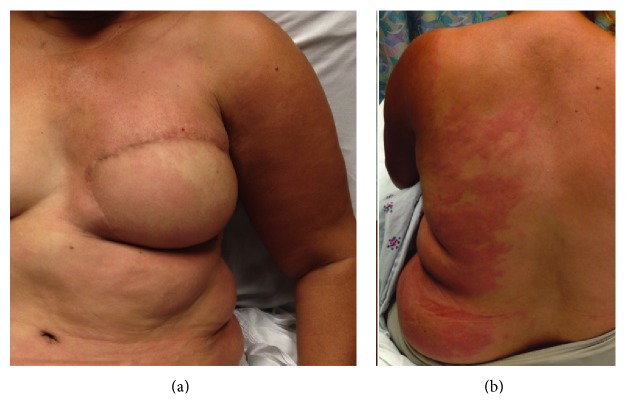
Lymphedema cellulitis.

A 46 year-old female with a history of left breast invasive ductal carcinoma (PT 4bN3a) with axillary metastasis presented with one day of fever, chills, and a painful rash over her left arm, breast, and back (Figures 1(a) and 1(b)). Her cancer treatment consisted of axillary lymph node dissection two years prior and a breast TRAM flap reconstruction without implant. She received four cycles of Adriamycin and Cytoxan and twelve cycles of taxol in addition to radiotherapy, which was completed more than a year prior to her presentation to the ED. She was currently taking tamoxifen.

Examination revealed erythematous, warm, indurated skin over the left upper arm, chest, abdomen, and left back that did not cross the midline aside from a small portion below the umbilicus. Exam was not significant for a fluctuant mass. A bedside ultrasound did not show a focal fluid collection, or a deep venous thrombosis in her upper extremity. Her vital signs were a temperature of 39.5 C; heart rate of 105 beats per minute; and a respiratory rate of 25 breaths per minute. Serum laboratory values were significant for a WBC of 20.6 K/cumm with 94.9% neutrophils, a C-reactive protein of 145.5 mg/dL, and an erythrocyte sedimentation rate (ESR) of 49 mm/hr.

Initial differential diagnosis included cellulitis, drug eruption, necrotizing soft tissue infection, erythroderma, cutaneous T-cell lymphoma, and toxic shock syndrome. A computed tomography (CT) scan of the chest and abdomen with intravenous (IV) contrast showed inflammation adjacent to the TRAM flap reconstruction site and in the subcutaneous tissues of the anterior abdominal wall.

She was diagnosed with lymphedema cellulitis in the ED and admitted to the hospital. She was started on IV ceftriaxone and vancomycin, which was continued for five days until she was transitioned to oral ciprofloxacin and trimethoprim-sulfamethoxazole. She was discharged on hospital day #5 and completed an additional 5-day course of the oral antibiotics. Punch biopsy showed perivascular and interstitial dermatitis with neutrophils. Blood and biopsy cultures were negative for bacterial growth. At her follow-up appointment, she reported complete resolution of her symptoms.

## 3. Discussion 

Approximately 200 million persons worldwide suffer from lymphedema, with filariasis being the most common cause [4]. Lymphedema affects women more than men and can range from subclinical impairment of lymphatic drainage to reversible pitting edema, irreversible brawny edema, or elephantiasis. In developed nations, lymphedema is predominantly caused by lymphadenectomy [4]. Axillary lymph node dissection remains the mainstay of staging and treatment planning in patients with breast cancer, and thus damage to and/or removal of the axillary lymph nodes is not uncommon [1]. Lymphedema acts as an ideal medium for bacterial growth, likely due to decreased lymphatic flow and impaired elimination of phagocytosed bacteria [1, 3]. Patients with cellulitis complicating lymphedema can have abrupt onset of symptoms and tend to have a longer duration of the inflammatory response, manifested by fevers, erythema at the site, and tachycardia [3, 5]. Lymphedema has also been found to be an independent risk factor for cellulitis treatment failure [2].

Patients who have undergone lymph node dissection alone are at increased lifelong risk for cellulitis, and those with lymphedema are at even higher risk [1, 6]. Additional risk factors include a history of a radical hysterectomy, mastectomy, radiation therapy, or lymphatic filariasis [3]. Patients who present status post lumpectomy and radiation, which is becoming increasingly more common as cancers are detected in earlier stages, tend to develop cellulitis earlier than those who have had a mastectomy or other more aggressive initial interventions, for reasons that remain unclear [1]. Increased risk for cellulitis is seen in those with more severe lymphedema, and also in those who develop lymphedema more than one year following surgery [1]. As was seen in our patient, lymphedema and cellulitis involving the ipsilateral trunk can also develop following mastectomy secondary to the lymphatic track it shares with the axilla [7].

Patients should be counseled to avoid trauma or blood draws to the affected extremity, employ frequent handwashing, and take measures to protect against skin breaks from daily activities such as handwashing with protective gloves and liberal moisturizing [6]. Sentinel lymph node biopsy instead of initial axillary lymph node dissection may also decrease the risk of lymphedema cellulitis from occurring as it has been associated with less lymphedema overall [8].

In addition to a complete blood count, procalcitonin and C-reactive protein (CRP) have been discussed in the literature as potentially useful lab values. Procalcitonin is a polypeptide hormone that is associated with infection and inflammation in patients. [9] CRP is an inflammatory marker that has been heavily investigated due to its potential to determine disease progress and effectiveness of treatment [10].

Treatment consists of antibiotics to cover gram-positive cocci, most commonly non-group A streptococcus [1, 8]. Blood cultures are typically negative, which is thought to be due to the large numbers of cytokines and lymphokines present in lymph [3, 11]. Early initiation of antibiotics is ideal, as cessation of bacterial multiplication may help prevent further damage to the lymphatic drainage system [3]. Additionally, because much of the skin reaction and fever is thought to be secondary to the antigenic response, anti-inflammatory medications may be of benefit [11]. Hospital admission is recommended for patients with signs of sepsis, who have failed outpatient oral antibiotics, or fail to respond to first or second line antibiotics [8]. Antibiotics should be continued until all signs of infection have resolved with a minimum course of 14 days [5]. Recommended initial oral regimens include amoxicillin and clindamycin as first and second line treatments, respectively. Initial IV antibiotic regimens should cover both streptococcus and staphylococcus species [5].

Many patients with lymphedema cellulitis will have recurrent episodes [1, 3, 5, 12, 13]. Long-acting depot penicillin or daily low-dose oral clindamycin or penicillin V has been suggested as prophylactic options, though penicillin use is limited by rising resistance [5, 14, 15]. Alternatively, decongestive lymphatic therapy has been shown to significantly reduce lymphedema and cellulitis recurrence [5, 12]. A recent study also showed a reduction in recurrent lymphedema cellulitis following lymphaticovenular anastomosis [13].

In addition to increased risk of infection, the immune dysfunction associated with lymphedema can lead to malignancy. The most commonly described malignancy is an angiosarcoma, commonly called Stewart-Treves syndrome, which presents as multiple reddish-blue macules or nodules. Additional associated malignancies include Kaposi sarcoma, basal cell carcinoma, and squamous cell carcinoma [16].

In conclusion, lymphedema cellulitis is a clinically important presentation to the emergency department. Our case represents a scenario of truncal and upper extremity lymphedema and infection following axillary lymph node dissection and mastectomy for breast cancer. In addition to rapid onset of symptoms and systemic response, patients with lymphedema cellulitis are at risk for prolonged symptoms, treatment failure, and recurrent episodes.

## References

[1] Simon M. S., Cody R. L. (1992). Cellulitis after axillary lymph node dissection for carcinoma of the breast. *The American Journal of Medicine*.

[2] Peterson D., McLeod S., Woolfrey K., McRae A. (2014). Predictors of failure of empiric outpatient antibiotic therapy in emergency department patients with uncomplicated cellulitis. *Academic Emergency Medicine*.

[3] Woo P. C. Y., Lum P. N. L., Wong S. S. Y., Cheng V. C. C., Yuen K. Y. (2000). Cellulitis complicating lymphoedema. *European Journal of Clinical Microbiology and Infectious Diseases*.

[4] Carlson J. A. (2014). Lymphedema and subclinical lymphostasis (microlymphedema) facilitate cutaneous infection, inflammatory dermatoses, and neoplasia: a locus minoris resistentiae. *Clinics in Dermatology*.

[5] Keely V., Mortimer P., Welsh J. (2013). *Consensus Document on the Management of Cellulitis in Lymphoedema*.

[6] Bertelli G., Dini D., Forno G. G., Gozza A. (1994). Preventing cellulitis after axillary lymph node dissection. *The American Journal of Medicine*.

[7] Roberts C. C., Levick J. R., Stanton A. W. B., Mortimer P. S. (1995). Assessment of truncal edema following breast cancer treatment using modified Harpenden skinfold calipers. *Lymphology*.

[8] Husted Madsen A., Haugaard K., Soerensen J. (2008). Arm morbidity following sentinel lymph node biopsy or axillary lymph node dissection: a study from the Danish Breast Cancer Cooperative Group. *The Breast*.

[9] Oh C., Pang S. M., Chlebicki M. P., Ho Z., Thirumoorthy T. (2012). Cellulitis: making the right diagnosis and its management: a Singapore experience. *Hong Kong Journal of Dermatology and Venereology*.

[10] Pitsavos C., Panagiotakos D. B., Tzima N. (2007). Diet, exercise, and C-reactive protein levels in people with abdominal obesity: the ATTICA Epidemiological Study. *Angiology*.

[11] Martinez E., Marcos A., Domingo P. (1994). Cellulitis after axillary lymph node dissection. *The American Journal of Medicine*.

[12] Baddour L. M. (2001). Recent considerations in recurrent cellulitis. *Current Infectious Disease Reports*.

[13] Mihara M., Hara H., Furniss D. (2014). Lymphaticovenular anastomosis to prevent cellulitis associated with lymphoedema. *British Journal of Surgery*.

[14] Chlebicki M. P., Oh C. C. (2014). Recurrent cellulitis: risk factors, etiology, pathogenesis and treatment. *Current Infectious Disease Reports*.

[15] Klempner M. S., Styrt B. (1988). Prevention of recurrent staphylococcal skin infections with low-dose oral clindamycin therapy. *The Journal of the American Medical Association*.

[16] Lee R., Saardi K. M., Schwartz R. A. (2014). Lymphedema-related angiogenic tumors and other malignancies. *Clinics in Dermatology*.

